# Commentary by Spear, R. on “Integration of Water, Sanitation, and Hygiene for the Prevention and Control of Neglected Tropical Diseases: A Rationale for Inter-Sectoral Collaboration:” Can the Control of NTDs Profit from a Good WASH?

**DOI:** 10.1371/journal.pntd.0002473

**Published:** 2013-09-26

**Authors:** Robert C. Spear

**Affiliations:** Center for Occupational and Environmental Health, University of California, Berkeley, California, United States of America; George Washington University, United States of America

The deliberations of the working group presented in this report offer an interesting and timely summary that again raises issues that have long been apparent to many of us who work on the control of neglected tropical diseases. In short, the central issue they address is how best to integrate the widespread use of chemotherapeutic drugs administered to human and/or animal populations with more sustainable, but usually more expensive, environmental modifications to reduce disease transmission. The evidence from the field suggests that we have ineffectively marketed the need for an integrated approach both to ourselves and to policy makers within and outside the public health world. Many of the key issues and challenges are well articulated in the report, including capacity building and training that are increasingly necessary in an era of rapidly changing tools for disease surveillance as well as for control.

However, there is one concept that lies at the intersection of the NTD and WASH worlds that is not mentioned, but not surprisingly so, since it is not common to either. It is the central role of human exposure to pathogens in the environment, not as a vague concept or a necessary event in the infection process, but as a predictive or evaluative measure of infection risk. For environmentally mediated infectious diseases that are caused by specific etiological agents, exposure assessment can provide a powerful tool in the design, targeting, and evaluation of control interventions. This is the case for many NTDs and is in contrast to the often diverse agents and pathways of exposure responsible for the diarrheal diseases that are a major focus of the WASH world.

The need for the exposure concept arises in the several places in the report that refer to the lack of evidence for the effectiveness of WASH interventions and where randomized control trials are implied as the preferred means of providing widely useful evidence. I argue that to study environmental interventions to control NTDs, randomized control trials have a role to play in answering very specific questions but, without accompanying exposure-relevant studies, they are likely to provide only partial information that is difficult to extrapolate [1]. This is because of the heterogeneity in both space and time that characterizes human exposures to hazardous agents. In the case of chemical and physical agents, this large variability in exposure extends from well-controlled indoor environments typical of the manufacturing industries [2] to much more variable outdoor environments that are subject to weather and/or ecological effects and have much in common with those in which pathogen transmission takes place [3], [4]. In many fields concerned with the control of environmental exposures to hazardous agents, some level of exposure assessment precedes and informs the selection and implementation of a particular control strategy [5]. That is, effective environmental controls are recognized to depend on identification of the pathways by which exposure occurs and an assessment of their relative importance and susceptibility to control. In the community setting these pathways are often both host and site specific.

To exemplify the exposure-based perspective in the context of NTDs, suppose we are concerned with minimizing parasite burden in an individual as a result of environmental exposure. At time *T* the burden is *w*(*T*) = *αE*(T)+*w*_0_ where *w*_0_ is the burden at *T = 0, E(T)* the cumulative exposure to the parasite in environmental media from *0* to time *T*, and *α* the fraction of parasites incident on body barriers that mature into adult parasites *in vivo*. Each of the variables on the right of the equation can vary a great deal from person to person, even within a small and geographically concentrated population [6]. If the population has previously been treated through an MDA program, drug compliance and efficacy determine the distribution of *w*_0_ and the distribution of *α* depends on individual susceptibility including vaccine coverage and effectiveness, if a vaccine exists. While data on *w*_0_ can be obtained for some infectious agents and *α* is of current interest to our group, for example, in the context of surveillance for schistosomiasis in the low transmission environment [7], *E(t)* is the issue when environmental controls are being considered.

A basic question related to *E(t)* concerns identifying pathways of exposure experienced by the population of interest. Again, schistosomiasis presents a good example because the exposure is only via contact with surface waters susceptible to schistosome contamination. In irrigated agricultural settings, the waters of interest are principally in irrigation ditches and small streams which harbor the snail intermediate hosts. Hence, there is another exposure is of interest, that of the snail whose miricidial exposure and infection results in the cercarial concentrations subsequently infective to the mammalian host. The exposure and infection of snails as well as that of humans varies widely in both space and time. The inherent complexity of the transmission cycle makes it immediately clear why it is desirable to develop interventions that are effective yet insensitive to the variability inherent in these processes, MDA being the prime example. Effective environmental interventions are also possible if the interventions are multifaceted and applied at a large scale, as is the case now for schistosomiasis in China which is benefitting from massive investments in rural development only secondarily motivated by disease control considerations.

In the absence of large-scale rural development, however, often the variability in exposure and infection of vectors and hosts in space and time does matter in achieving effective control. For example, for infectious agents where *E(t)* is driven by the prevalence or intensity of infection within an isolated community, the local environmental determinants of the basic reproductive number, *R_0_*, are central [8]. Alternatively, if a village's *R_0_* is less than unity, connectivity via host mobility or transport in environmental media may be the crucial determinants of the spatial scale of effective control [9], [10].

Assessing the concentrations of hazardous agents in environmental media is generally central to exposure assessment. For example, the world of waterborne pathogens is replete with water quality standards which define contaminant limits and measurement methods for various uses from drinking water to crop irrigation [11]. For some NTDs, methods for environmental assessment of human infection risk are available and rely, for example, on estimating the spatial and temporal distribution of disease vectors. Also methods for assessing the spatial dimensions of exposure using GIS and GPS technology have been used and are available for broader application to NTDs [12]. However, methods for assessing the environmental concentration or density of the biological agents causing NTDs are truly neglected. There are many NTDs for which methods for environmental measurements relevant to exposure assessment have not been developed or may not be widely available. More research is needed in this area to examine the degree to which practical measures of exposure can reliably and sufficiently quantify the risk of infection in real-world settings. New and robust approaches for sampling waterborne, foodborne, and soil-associated agents, in particular, are needed, but are largely unexplored.

Clearly, an exposure-based approach to the control of NTDs has its own range of challenges. But at the intersection of disease control strategies for NTDs and the WASH world, there are approaches to the selection, design, and implementation of environmental controls that utilize exposure assessment methods that can provide a useful bridge as they have so successfully in other arenas.
